# The offering of family presence during resuscitation: a systematic review and meta-analysis

**DOI:** 10.1186/s40560-015-0107-2

**Published:** 2015-10-14

**Authors:** Simon JW Oczkowski, Ian Mazzetti, Cynthia Cupido, Alison E. Fox-Robichaud

**Affiliations:** Department of Medicine, Division of Critical Care, McMaster University, Hamilton, Canada; Department of Critical Care, Schulich School of Medicine and Dentistry, Western University, London, Canada; Department of Pediatrics, Division of Critical Care, McMaster University, Hamilton, Canada; Hamilton General Hospital, McMaster Clinic, 4th floor, Room 434, 237 Barton St East, Hamilton, ON L8L 2X2 Canada

**Keywords:** Resuscitation, Family, Family-centered care, Family presence

## Abstract

**Background:**

Family members may wish to be present during resuscitation of loved ones, despite concerns that they may interfere with the resuscitation or experience psychological harm.

**Methods:**

We conducted a systematic review to determine whether offering family presence during resuscitation (FPDR) affected patient mortality, resuscitation quality, or family member psychological outcomes. We searched multiple databases up to January 2014 for studies comparing FPDR to usual care. Two reviewers independently assessed eligibility, risk of bias, and extracted data. Data from randomized controlled trial (RCTs) at low or uncertain risk of bias were eligible for pooling. Quality of evidence was assessed using GRADE.

**Results:**

Three RCTs evaluated the offering of FPDR in adults, finding no differences in resuscitation duration, prehospital/emergency room mortality (odds ratio [OR] 0.80, 95 % confidence interval [CI] 0.54–1.19), or 28-day mortality (OR 1.24, 95 % CI [0.50–3.03]). Hospital Anxiety and Depression Scale scores for anxiety (mean difference [MD] −0.99, 95 % CI [−1.77, −0.22]) and depression (MD −1.00, 95 % CI [−1.78, −0.23]), along with Impact of Events Scale intrusion score (MD −1.00, 95 % CI [−1.96, −0.03]), were better in family members offered FPDR. One RCT evaluated FPDR in pediatric patients, finding no mortality differences at 28 days (OR 0.30; 95 % CI [0.11–0.79]), but did not report psychological outcomes in family members.

**Conclusions:**

Moderate-quality evidence suggests the offering of FPDR does not affect adult resuscitation outcomes and may improve family member psychological outcomes. Low-quality evidence suggests FPDR does not affect pediatric resuscitation outcomes. The generalizability of these findings outside the prehospital and emergency room setting is limited due to the absence of trials in other health care settings.

**Electronic supplementary material:**

The online version of this article (doi:10.1186/s40560-015-0107-2) contains supplementary material, which is available to authorized users.

## Background

### Rationale

Closed-chest compressions were first used for cardiac arrest in the 1960s, leading to the publication of the first cardiopulmonary resuscitation (CPR) guidelines in 1966 [1]. The introduction of bystander CPR in the first advanced cardiac life support courses in 1976 were followed shortly thereafter by reports of family presence during resuscitation (FPDR) [1, 2]. Survey data from these foundational reports suggested that while family members were often receptive to the idea of being present during resuscitation, health care providers were often averse to the practice, citing concerns that family members’ presence would adversely affect the patient’s outcomes from resuscitation [2]. Although early experiences for FPDR were in the context of CPR, “resuscitation” can refer to other acute, life-threatening situations, such as trauma or treatment of shock.

This has been a contested issue, with many published perspectives for and against FPDR, summarized in a recent narrative review [3]. In favor of FPDR is the provision to the family an understanding of what it means to “do everything,” the closure family members obtain when they are guided through the process, and orchestration of the best death possible, when death is inevitable [3]. Those opposed to FPDR often cite concerns about interference with resuscitation efforts and repercussions to the health care team, as well as psychological trauma to family members, such as depression or post-traumatic stress disorder (PTSD) [3].

Two recent systematic reviews have evaluated family and health care provider support for FPDR [4, 5]. McAlvin and Carew-Lyons reviewed six studies, including one prospective observational study, one qualitative study, and four retrospective mixed-methods studies, concluding that FPDR may improve family satisfaction and coping [4]. Porter et al. identified 14 studies, including 9 surveys, 2 observational studies, and 1 randomized controlled trial (RCT), finding FPDR to be supported by families and staff [5]. Though these reviews found FPDR to be an acceptable practice, neither study specifically evaluated the quality of evidence for other patient- and family-important outcomes, including patient mortality, resuscitation quality, and the long-term psychological effects upon family members, which are often the focus of clinician concern. To evaluate these claims, we conducted a systematic review and meta-analysis of studies evaluating the effect of offering FPDR compared to usual care on patient mortality, resuscitation quality, and the psychological health of family members. Our structured research question was as follows:

For families of patients (adult or pediatric) undergoing resuscitation, does being present (or being systematically offered the opportunity to be present) during the resuscitation, versus not being present (or not being systematically offered the opportunity to do so), affect patient mortality, quality of resuscitation, or the psychological health of family members?

## Methods

### Study eligibility criteria

Our initial inclusion criteria included RCTs published or in journals or abstract form, in any language, comparing the offering of family presence during resuscitation versus usual care. “Family” included individuals who were biologically related, spouses, or close friends. Unpublished studies registered in clinical trial databases or on the Internet were also sought. We included studies with patients receiving resuscitation for shock, cardiac arrest, or trauma. Studies evaluating family presence during invasive procedures outside the context of resuscitation were excluded. Both adult and pediatric studies were included in the search; however, the studies were analyzed separately (Table 1).

**Table 1:** Study eligibility criteria and study outcomes

Study type	• Randomized controlled trials
	• Published in journals or abstract form
	• No date restriction
	• No language restriction
Population	• Patients undergoing resuscitation, as defined by study authors, including cardiac arrest, respiratory arrest, or circulatory failure/shock, and trauma
	• Resuscitation occurring in the outpatient/community, inpatient ward, or intensive care unit settings
Intervention	• Family presence during resuscitation or systematic offering of family presence during resuscitation
	• May or may not include presence of support staff during resuscitation, presence of follow-up/debriefing with family, or referral to counseling following resuscitation
Comparison	• Usual care, family not present during resuscitation, or not systematically offered the opportunity to be present
	• May or may not include presence of support staff during resuscitation, presence of follow-up/debriefing with family, or referral to counseling following resuscitation
Primary outcomes	• Patient outcomes: mortality, quality of resuscitation (duration of resuscitation, number, and timing of critical resuscitation events)
	• Family outcomes: symptoms of depression, symptoms of anxiety

### Information sources, search strategy, and study selection

MEDLINE, Embase, Cochrane Central Register of Controlled Trials, CINAHL, clinicaltrials.gov, and Google Scholar were searched using computerized search protocols from database inception up to August 2015 (electronic search strategies are included in Additional file 1). The references of articles reviewed for eligibility were hand-searched in duplicate for further potentially relevant articles. Study investigators, study time period, population characteristics, and study methodology were closely examined to ensure that multiple reports of the same experimental data were not included (Table 2). Two reviewers (SO, IM) independently assessed study eligibility using standardized, piloted eligibility forms. Provision was made for arbitration by a third co-investigator (AFR) in the event of disagreement between the reviewers about study eligibility.

**Table 2:** Description of included studies

Author, year	Study design	Study location	Sample size	Population description	Study intervention	Primary outcomes reported	Other findings	Risk of bias assessment
Adult studies								
Jabre et al. 2013 [9]	Cluster RCT	France	FPDR 266, 304 control	570 relatives of patients in cardiac arrest, including traumatic arrest	Systematically offering family opportunity to be present during CPR with chaperone vs. usual care	• Patient mortality (prehospital/ER and 28 day)		Low risk of bias (Cochrane)
						• Duration of resuscitation		
						• Family member symptoms of anxiety (3 months)		
						• Family member symptoms of depression (3 months)		
Holzhauser et al. 2006 [11]	Single-center RCT	Australia	FPDR 60, 39 control	Adult family members of adult, non-trauma patients undergoing resuscitation in the emergency department	Family members randomized in 2:1 fashion to systematic offering of FPDR with chaperone vs. usual care	• patient mortality (prehospital/ER)	Association between family members who participated in FPDR and belief that their presence was beneficial to the patient	Low risk of bias (Cochrane)
Robinson et al. 1998 [10]	Single-center RCT	UK	13 FPDR, 12 usual care	Consecutive adult patients undergoing resuscitation in the emergency department for cardiac arrest or trauma	Randomized in 1:1 fashion to systematic offering of FPDR with accompaniment with a chaperone vs. no systematic offering of FPDR with chaperone	• Patient mortality (prehospital/ER)		Moderate risk of bias (Cochrane)
						• Family member symptoms of anxiety (3 months, 9 months)		
						• Family member symptoms of depression (3 months, 9 months)		
Pediatric studies								
Dudley et al. 2009 [12]	RCT	USA	283 intervention; 422 control	1229 pediatric patients undergoing trauma resuscitation; 283 witnessed resuscitation; 422 did not	Families on even days randomized to systematic offering of family presence during trauma resuscitation with trained social workers as support personnel vs. waiting outside of trauma room with supportive social worker present	• Patient mortality (hospital discharge)	No differences in success rate of critical interventions. Health care providers surveyed believe there was minimal effect on resuscitation. Families surveyed were strongly supportive and believed their presence to be beneficial to the patients	High risk of bias (Cochrane)
						• Duration of resuscitation		
						• Time until critical event (CT scan)		

*RCT* randomized controlled trial, *FPDR* family presence during resuscitation, *CPR* cardiopulmonary resuscitation, *ER* emergency room, *CT* computed tomography

### Data collection and data items

The same two reviewers independently extracted data in duplicate using piloted data collection forms. Our primary outcomes were patient mortality, quality of resuscitation (frequency and time until key resuscitation interventions, duration of resuscitation), and psychological outcomes of family members (symptoms of anxiety, depression, satisfaction with care). Any study data that was missing was sought from study authors using contact information listed on the study or found online using a simple web search (Table 1).

### Risk of bias in individual studies

Study quality was assessed with the tool used by the Cochrane Database of Systematic Reviews to determine the appropriateness of the random sequence generation, allocation concealment, blinding of participants and personnel, incomplete outcome data, and selective reporting [6]. Studies were assessed independently by both reviewers and reported as being at “high,” “low,” or “uncertain” risk of bias for each category, with provision for review by a third investigator in the event of disagreement.

### Synthesis of results and summary measures

Extracted data from eligible studies were entered into Revman™ v5.1 for data synthesis. For data presented as median and interquartile range, estimates of mean and standard deviation were determined using the method described by Hozo et al. [7], which would reduce the precision of our estimate of effect. Adjusted sample sizes were calculated for cluster randomized trials by taking into account the average cluster size and inter-cluster correlation coefficients. Statistical heterogeneity was assessed for each outcome of interest, and reported using *I*^2^, with values greater than 50 % indicating substantial heterogeneity. For outcomes not found to have significant heterogeneity, summarized outcomes (standardized mean difference for continuous variables or relative risk for dichotomous variables) and 95 % confidence intervals (CIs) were calculated using a random-effects model. For all tests, *p* values less 0.05 were considered to be statistically significant. Where possible, we performed sensitivity analysis by analyzing data on the principle of intention-to-treat (ITT) and per-protocol analysis. For ITT analysis, we compared patients randomized to the offering of FPDR to those randomized to usual care. For per-protocol analysis, we compared family members who actually were present during resuscitation to family members who were not present. For outcomes where FPDR was expected to be of benefit, such as psychological outcomes in family members, data from an ITT analysis is presented, as it is more likely to provide a conservative estimate of effect. For outcomes where FPDR may potentially be of harm, such as patient mortality, duration of resuscitation, or time to critical intervention, per-protocol data was used where available, as it gives a less conservative estimate of risk.

### Risk of bias across studies and across outcomes

Quality of evidence supporting each outcome was assessed using the Grading of Recommendations Assessment, Development and Evaluation (GRADE) approach. GRADE takes into account study risk of bias, publication bias, imprecision, inconsistency, and indirectness of the evidence [8]. GradePRO software (www.guidelinedevelopment.org) was used to generate summary of findings tables including data from RCTs or observational studies if no RCTs were available for outcomes of interest.

### Ethics

As this study was a systematic review of data from previously published studies, ethics board approval was neither required nor sought.

## Results

### Study selection

**Fig. 1:**
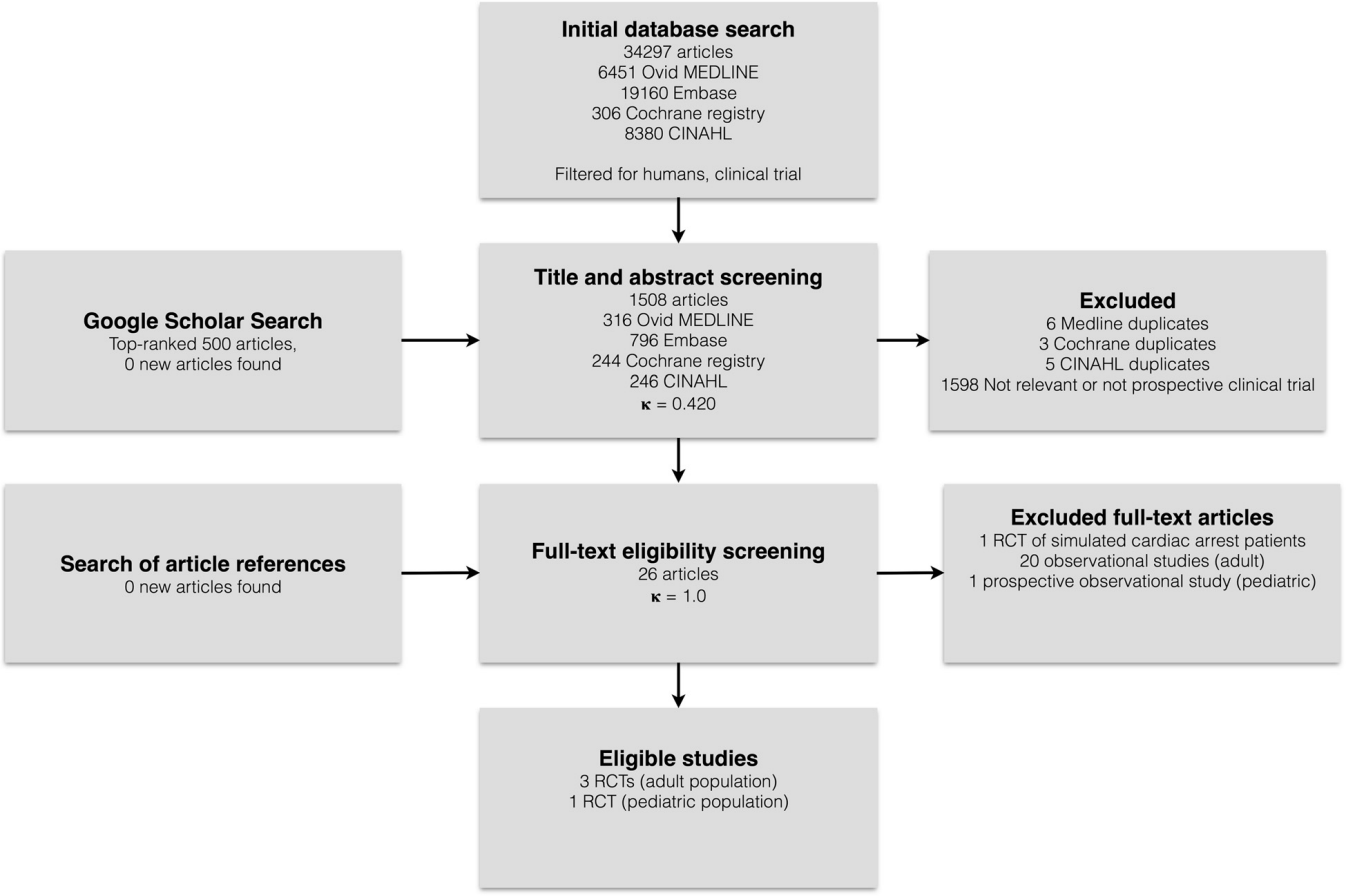
Flow chart showing screening, inclusion, and exclusion of retrieved studies; *RCT* randomized controlled trial

A total of 34,297 articles were retrieved in our initial search and filtered for human studies and clinical trials. Titles and abstracts of the remaining 1602 articles were screened in duplication. Twenty-six potentially relevant articles for FPDR in adults and children were found, with moderate agreement between reviewers (kappa = 0.420). Of these studies, three RCTs in adults, covering all of our predetermined outcomes of interest, met inclusion criteria, with perfect agreement between reviewers (kappa = 1.0). For pediatric studies, only one RCT, which did not present data on psychological outcomes for family members, met our eligibility criteria (Fig. 1).

### Study characteristics, results of individual studies, and risk of bias within studies

**Fig. 2:**
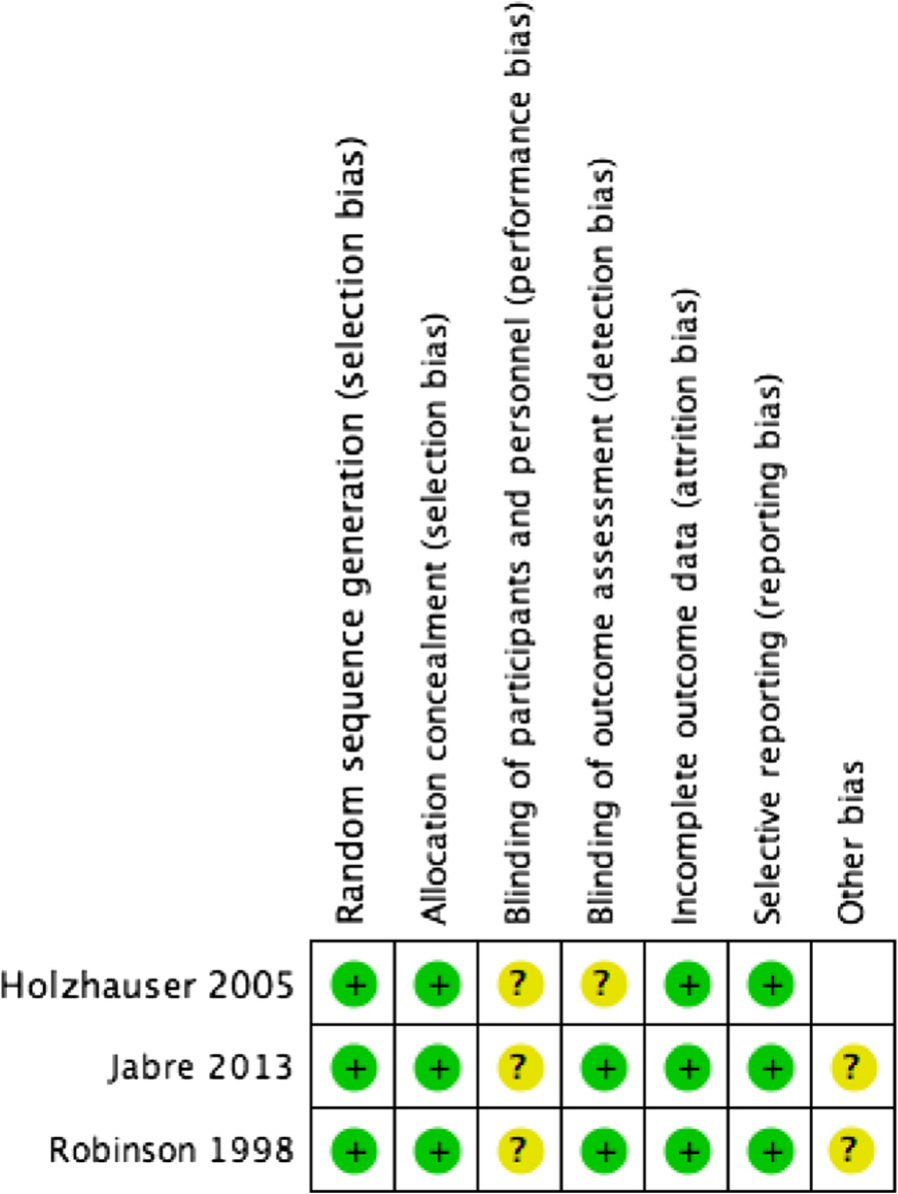
Risk of bias assessment for adult RCTs evaluating FPDR

Three RCTs compared the systematic offering of FPDR to usual care (Fig. 2). The largest, by Jabre et al., was a cluster RCT of 15 prehospital emergency medical units and included 570 families of patients undergoing resuscitation, judged to be at low risk of bias. No significant differences in mortality, duration of resuscitation, or resuscitation interventions were seen. Lower rates of PTSD-related symptoms and anxiety-related symptoms were seen at 90 days for family members randomized to FPDR [9]. In a small RCT at moderate risk of bias, Robinson et al. randomized 25 family members of patients undergoing resuscitation in the emergency room to FPDR versus usual care. No statistically significant differences in patient mortality or family PTSD or anxiety-related outcomes were seen at 3 or 9 months following the intervention. The trial was stopped early for perceived benefits to family members at the time of resuscitation with FPDR, though this outcome was not captured in the study’s outcome measures [10]. A third RCT by Holzhauer et al., considered to be at low risk of bias, randomized 88 family members of patients undergoing resuscitation in the emergency room (ER) in a 2:1 ratio to offering of FPDR versus usual care. It captured survival data, as well as qualitative data. No significant differences in resuscitation outcomes were seen [11]. In all three trials, families were accompanied by a trained chaperone into the resuscitation area. No RCTs evaluated FPDR in the inpatient or intensive care unit (ICU) setting (Table 2).

**Fig. 3:**
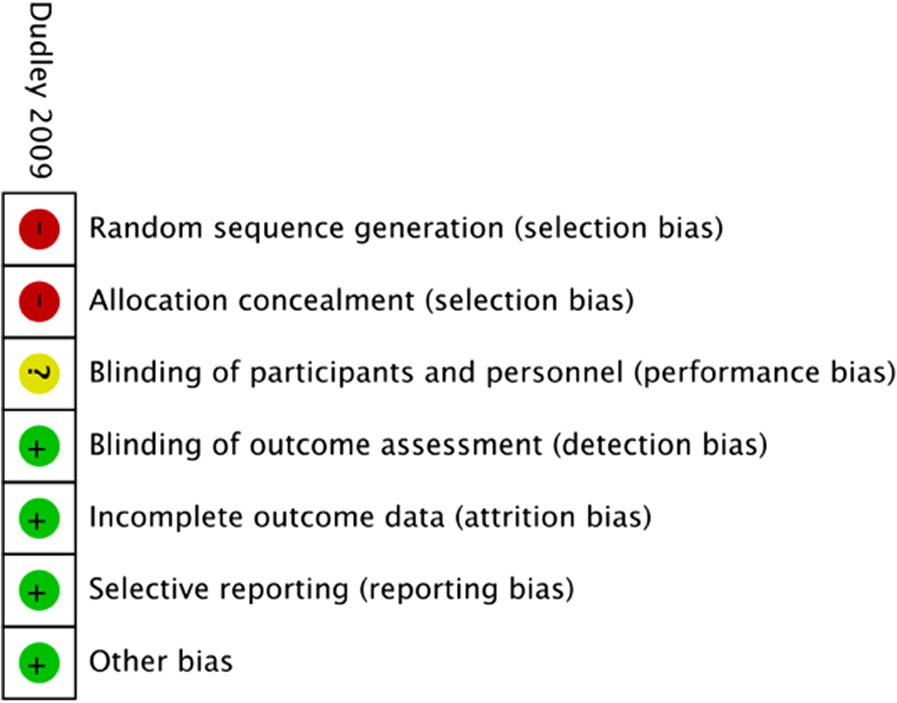
Risk of bias assessment for pediatric RCTs evaluating FPDR

Only one RCT by Dudley et al. [12] studied FPDR in a pediatric population and was judged to be at high risk of bias (Fig. 3). Of 1229 pediatric patients undergoing trauma resuscitation in the ER, 705 had family members present who were randomized to FPDR versus usual care on alternating even and odd days. Although limited by poor randomization (allocation according to day of enrollment), there were no differences between the groups for the time required for critical care intervention, success rates of interventions, duration of resuscitation, or time to CT scan (mean difference (MD) = 0; 95 % CI −2 to +2). There was a statistically significant reduction in mortality at 28 days (OR 0.30; 95 % CI = 0.11 to 0.79, *p* = 0.02) for patients assigned to FPDR. Health care providers surveyed agreed there was minimal effect on resuscitation. Families surveyed strongly believed their presence was beneficial to the patients, though no data on the psychological outcomes of family members were recorded [12].

### Synthesis of results and rating quality of evidence across studies

**Fig. 4:**
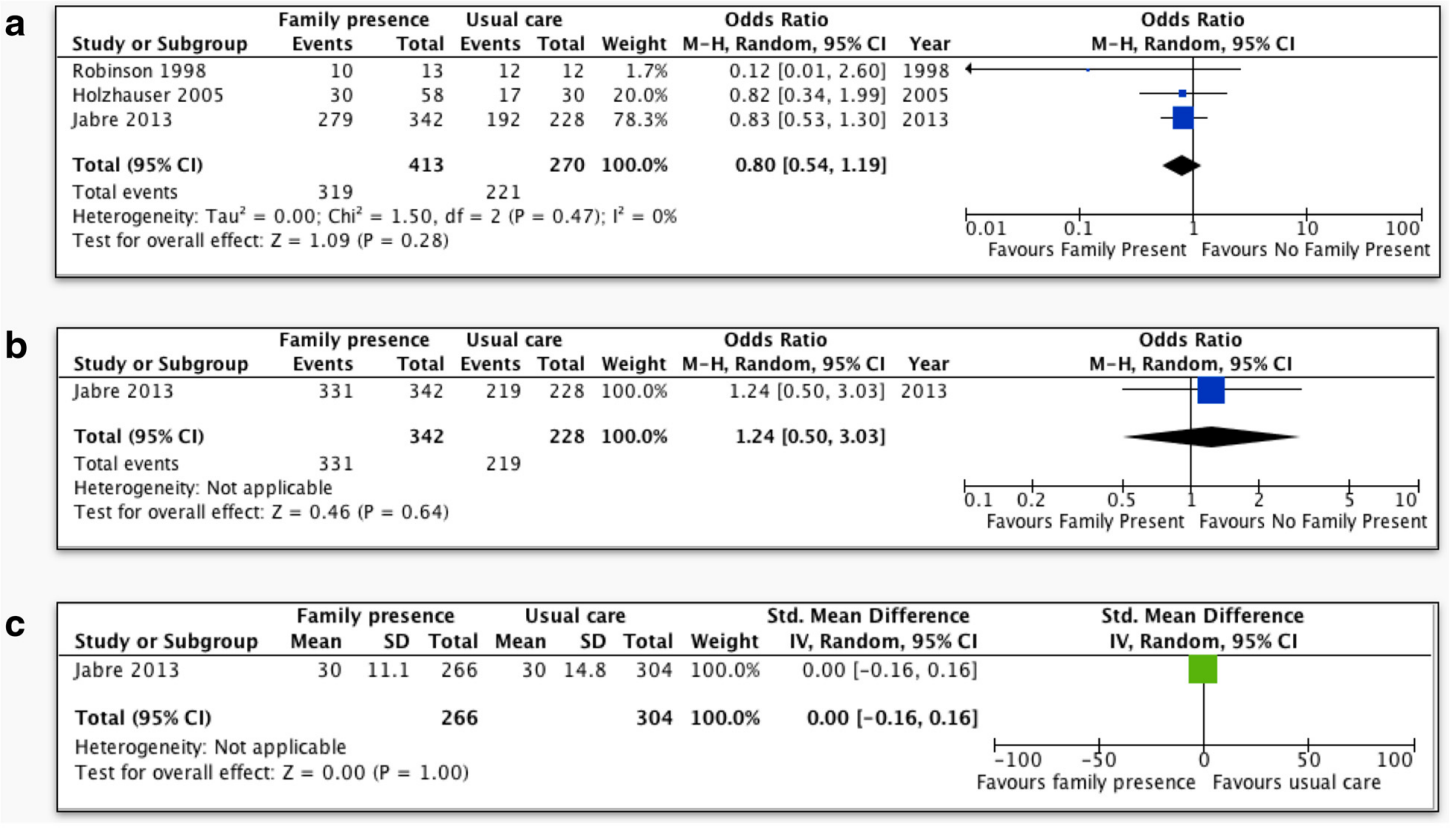
Patient outcomes for FPDR. **a** Prehospital/ER mortality (adult patients, per-protocol analysis); **b** mortality at 28 days (adult patients, per-protocol analysis); **c** duration of resuscitation (adult patients, per-protocol analysis); *CI* confidence interval

**Fig. 5:**
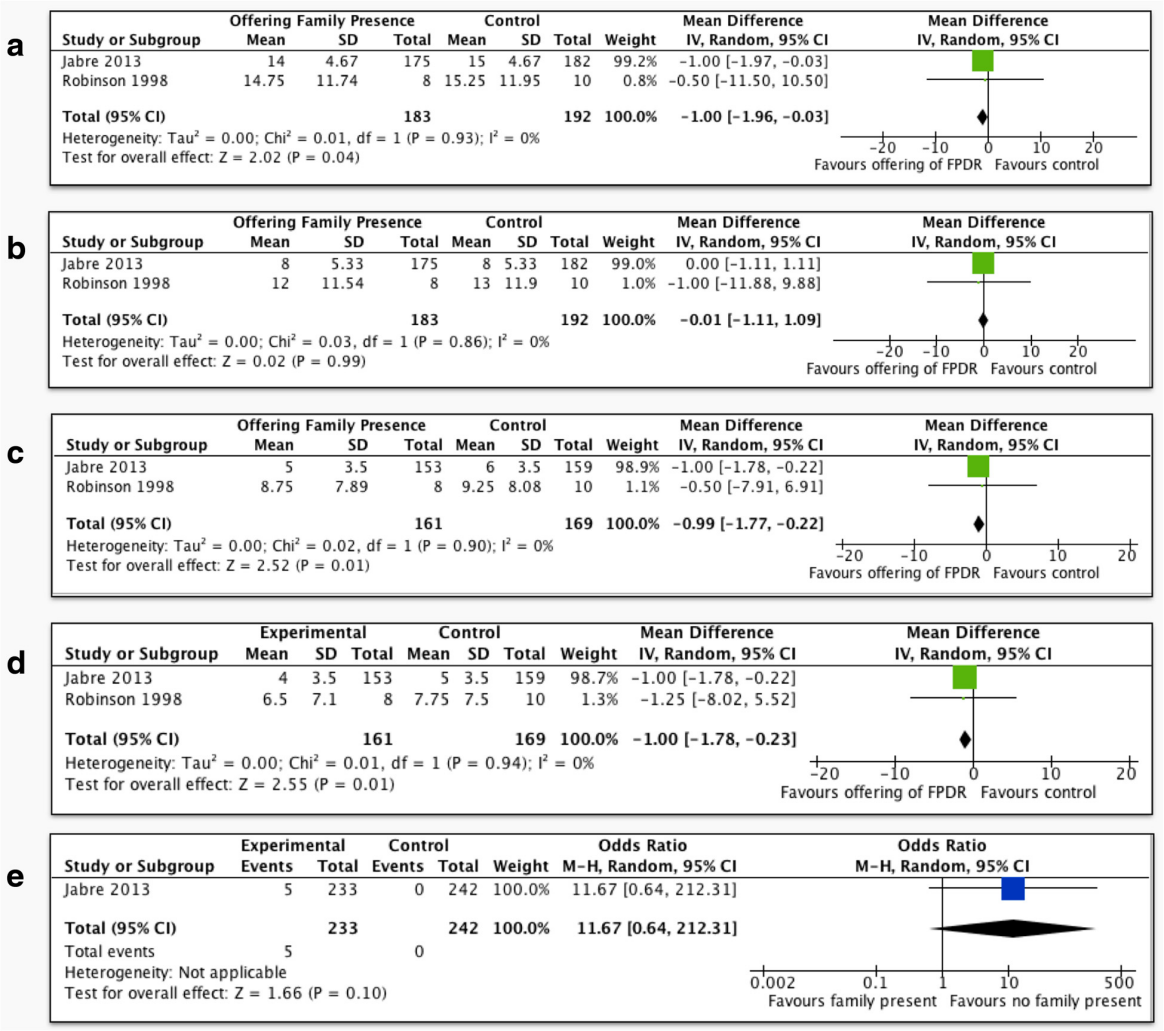
Family outcomes for FPDR. **a** Impact of Events Scale, intrusion subscale (adult patients, 90 days, intention-to-treat analysis); **b** Impact of Events Scale, avoidance subscale (adult patients, 90 days, intention-to-treat analysis); **c** Hospital Anxiety and Depression Scale, anxiety subscale (adult patients, 90 days, intention-to-treat analysis); **d** Hospital Anxiety and Depression Scale, depression subscale (adult patients, 90 days, intention-to-treat analysis); **e** suicide by family members (adult patients, 90 days, per-protocol analysis); *CI* confidence interval

Meta-analysis of the above three trials in adult patients [9–11] showed no statistically significant differences in prehospital and emergency room mortality (OR 0.80; 95 % CI 0.54 to 1.19, *p* = 0.28, Fig. 4a), mortality at 28 days (OR 1.24; 95 % CI 0.50 to 3.03, *p* = 0.64, Fig. 4b), or duration of resuscitation (mean difference = 0.0; 95 % CI −0.16 to 0.16 *p* = 1.00, Fig. 4c). Two RCTs, including 375 patients [9, 10], assessed PTSD-related symptoms using the Impact of Events Scale (IES), which scores 15 items from 0 to 5, up to a total score of 75, with a cutoff score of 33 indicating a probable diagnosis of PTSD [13]. FPDR was associated with lower scores in the intrusion (MD = −1.0; 95 % CI −1.96 to −0.03, *p* = 0.04, Fig. 5a) but not the avoidance subscale (MD −0.01; 95 % CI −1.11 to 1.09, *p* = 0.99, Fig. 5b). These two studies also assessed anxiety and depression-related symptoms using the Hospital Anxiety and Depression Scale (HADS), which scores each symptom on a scale of 0–21, with scores greater than 8 being cut points for caseness of anxiety or depression [14]. FPDR was associated with lower scores in the anxiety (MD = −0.99; 95 % CI −1.77 to −0.22, *p* = 0.01, Fig. 5c) and depression subscales (MD −1.0; 95 % CI −1.78 to −0.23, *p* = 0.01, Fig. 5d). There was a non-significant trend towards higher rates of suicide by those family members who had witnessed resuscitation when the data were analyzed on a per-protocol basis, but this trend was not present on ITT analysis (Fig. 5e). There was no evidence of statistical heterogeneity in any of the above comparisons. Using GRADE, outcomes based on RCTs start as “high”-quality evidence; however, we downgraded the quality of evidence for all outcomes due to imprecision because of the need to account for clustering effects in the study by Jabre et al. and the need to estimate mean and standard deviation from median and interquartile range. There was no evidence of inconsistency, or imprecision, and an insufficient number of studies to assess for publication bias (Table 3).

**Table 3:** Systematic offering of family presence compared to usual care for families of adult patients undergoing resuscitation

Outcomes	No. of participants (studies) follow-up	Quality of the evidence (GRADE)	Relative effect (95 % CI)	Anticipated absolute effects^a^	
				Risk with usual care	Risk difference with systematic offering of family presence
Prehospital and emergency department mortality	683 (3 RCTs)	⨁⨁⨁◯ Moderate^c^	OR 0.80 (0.54 to 1.19)	Study population	
				819 per 1000	36 fewer per 1000 (110 fewer to 24 more)
Mortality at 28 days	570 (1 RCT)	⨁⨁⨁◯ Moderate^c^	OR 1.24 (0.50 to 3.03)	Study population	
				961 per 1000	7 more per 1000 (36 fewer to 26 more)
Duration of Resuscitation	570 (1 RCT)	⨁⨁⨁◯ Moderate^b^	-	The mean duration of resuscitation in the control group was 30 min	Median 0 higher (0.01 lower to 0.01 higher)
Tine to key intervention assessed with: CT scan (trauma arrest) or first shock (cardiac arrest)	570 (1 RCT)	⨁⨁⨁◯ Moderate^c^	-	The mean time to key intervention in the control group was 18 min	The mean time to key intervention in the intervention group was 3 lower (1.2 lower to 0.85 lower)
Symptoms of anxiety in family members assessed with: Hospital Anxiety and Depression Scale (HADS) follow-up: 3 months	330 (2 RCTs) 3 months	⨁⨁⨁◯ Moderate^b^	-	The mean symptoms of anxiety in family members in the control group was 6.19	MD 0.99 lower (1.77 lower to 0.22 lower)
Symptoms of depression in family members assessed with: Hospital Anxiety and Depression Scale (HADS) follow-up: 3 months	330 (2 RCTs) 3 months	⨁⨁⨁◯ Moderate^b^	-	The mean symptoms of depression in family members in the control group was 5.16	MD 1 lower (1.78 lower to 0.23 lower)
Symptoms of PTSD (intrusion) assessed with: Impact of Events Scale (IES) follow-up: 3 months	375 (2 RCTs) 3 months	⨁⨁⨁◯ Moderate^b^	-	The mean symptoms of post-traumatic stress disorder in the control group was 15.01	MD 1 lower (1.96 lower to 0.03 lower)
Symptoms of PTSD (avoidance) assessed with: Impact of Events Scale (IES) follow-up: 3 months	375 (2 RCTs) 3 months	⨁⨁⨁◯ Moderate^b^	-	The mean symptoms of post-traumatic stress disorder in the control group was 8.26	MD 0.01 lower (1.11 lower to 1.09 higher)
Suicide attempts by family members follow-up: 9 months	465 (1 RCT) 9 months	⨁⨁⨁◯ Moderate^c^	OR 7.08 (0.39 to 128.79)	Study population	
				0 per 1000	0 fewer per 1000 (0 fewer to 0 fewer)

*CI* confidence interval, *RCT* randomized controlled trial, *RR* risk ratio, *OR* odds ratio, *CT* computed tomography

GRADE working group grades of evidence

High quality: We are very confident that the true effect lies close to that of the estimate of the effect

Moderate quality: We are moderately confident in the effect estimate: The true effect is likely to be close to the estimate of the effect, but there is a possibility that it is substantially different

Low quality: Our confidence in the effect estimate is limited: The true effect may be substantially different from the estimate of the effect

Very low quality: We have very little confidence in the effect estimate: The true effect is likely to be substantially different from the estimate of effect

^a^The risk in the intervention group (and its 95 % confidence interval) is based on the assumed risk in the comparison group and the relative effect of the intervention (and its 95 % CI)

^b^Unable to precisely estimate mean difference; data reported as median and IQR (presumed skewed data)

^c^Wide confidence intervals crossing the line of no effect

We were unable to conduct a meta-analysis in the pediatric setting due to the lack of published data. Only one randomized controlled trial [12] in the ER trauma bay compared FPDR to usual care for pediatric patients, demonstrating no evidence of interference by family members resulting in changes in time or success of major interventions, imaging procedures, or mortality. The serious risk of bias due to poor randomization in Dudley et al. [12] and the absence of any large effect size, dose response, or plausible residual confounding which would support rating up the quality of evidence resulted in a “low” quality of evidence for all outcomes. (Table 4).

**Table 4:** Systematic offering of family presence compared to usual care for families of pediatric patients undergoing resuscitation

Outcomes	No. of participants (studies) follow-up	Quality of the evidence (GRADE)	Relative effect (95 % CI)	Anticipated absolute effects^a^	
				Risk with usual care	Risk difference with systematic offering of family presence
Mortality prior to 28 days or discharge	705 (1 RCT)	⨁⨁◯◯ Low^bc^	OR 0.30 (0.11 to 0.79)	Study population	
				57 per 1000	39 fewer per 1000 (50 fewer to 11 fewer)
Duration of resuscitation	705 (1 RCT)	⨁⨁◯◯ Low^bc^	-	The mean duration of resuscitation in the control group was 15 min	Median 0 higher (1 lower to 1 higher)
Time to key intervention assessed with: CT scan (trauma arrest) or first shock (cardiac arrest)	705 (1 RCT)	⨁⨁◯◯ Low^bc^	-	The mean time to key intervention in the control group was 21 min	Median 0 higher (2 lower to 2 higher)

*CI* confidence interval, *RCT* randomized controlled trial, *RR* risk ratio, *OR* odds ratio, *CT* computed tomography

GRADE Working Group grades of evidence

High quality: We are very confident that the true effect lies close to that of the estimate of the effect

Moderate quality: We are moderately confident in the effect estimate: The true effect is likely to be close to the estimate of the effect, but there is a possibility that it is substantially different

Low quality: Our confidence in the effect estimate is limited: The true effect may be substantially different from the estimate of the effect

Very low quality: We have very little confidence in the effect estimate: The true effect is likely to be substantially different from the estimate of effect

^a^The risk in the intervention group (and its 95 % confidence interval) is based on the assumed risk in the comparison group and the relative effect of the intervention (and its 95 % CI)

^b^Study randomization on basis of even/odd days rather than on an individual basis

^c^Study included only pediatric patients undergoing trauma resuscitation; no children with primarily cardiac arrest were included

## Discussion

In adult populations, three RCTs have compared the offering of FPDR to usual care in the ER pre-ICU environment. The summarized evidence is of moderate quality and suggests that offering FPDR does not affect patient mortality or resuscitation quality. Moderate-quality evidence also suggests that offering FPDR can reduce symptoms of anxiety and depression in family members. No such evidence exists for patients in the inpatient ward or ICU settings. Of note, while there is a statistically non-significant trend towards increased risk of suicide amongst family members who witnessed resuscitation in the per-protocol analysis, this trend is not present on analysis by ITT. There is insufficient evidence to conclude the association is causal. It may be that family members who are more prone to suicide are also more likely to request or accept the opportunity to be present during resuscitation when offered. Systematically offering families the opportunity to be present may provide an opportunity to identify these high-risk individuals and prevent these adverse events. In the pediatric population, one RCT and one prospective cohort study have evaluated the offering of FPDR during trauma resuscitation in the ER. Both studies are of low quality. The summarized evidence, also of low quality, suggests that the offering of FPDR does not affect the quality or outcome of resuscitation. The concern of parental interference with the delivery of care was not borne out in either study. The study did not collect data on the psychological effects of offering FPDR to family members (Table 4).

Our systematic review is limited by the small number of included trials and their size and quality. The only large, high-quality RCT, published by Jabre et al., limited our meta-analysis due to its cluster-randomized design, which required us to take into account the average cluster size and clustering effects, reducing the precision of our estimated effects and resulting in a reduction to moderate-quality evidence, despite an otherwise excellent study design [10]. The clinical significance of small reductions in HADS and IES scores is unknown, although it is reassuring that the signal for family members offered FPDR is towards benefit rather than harm. Unfortunately, none of the pediatric trials we found systematically studied or reported the psychological outcomes of family members. Insufficient evidence existed to explore through subgroup analysis whether the effects of offering FPDR vary based on the setting or type of resuscitation (with vs. without CPR, traumatic vs. non-traumatic resuscitation); however, the adult studies evaluate resuscitation events with CPR, while the pediatric trial evaluated traumatic resuscitation (with or without CPR), and in both sets of studies, patient outcomes did not appear to be adversely affected.

As well, the generalizability of our study to other areas of the hospital may be limited as all of the trials took place in prehospital and ER settings. Given the infrequent nature of cardiac arrest, and the relative rarity of family member presence at the time of arrest in the ICU or ward, it seems unlikely that large, high-quality studies similar to Jabre et al. will be forthcoming in other settings in either the pediatric or adult population. Finally, none of the studies in adult patients included family members younger than 18 years of age. It is unclear whether these results are generalizable to a younger population of family members.

The strengths of our systematic review include its comprehensive literature search using multiple databases of the medical and nursing literature; its rigorous screening, quality assessment, and data extraction in duplicate by two separate authors using piloted forms; and the use of GRADE to evaluate the quality of evidence for each of our outcome of interest. Compared to earlier reviews on this topic [4, 5], our review is the only one to systematically review the important outcomes of patient mortality and the mental health outcomes of family members.

## Conclusions

There is limited evidence evaluating the effects of offering FPDR upon resuscitation outcomes and the psychological health of family members. However, the studies conducted to date provide moderate-quality evidence in adults, and low-quality evidence in children, that offering FPDR does not affect resuscitation outcomes. Moderate-quality evidence in adults suggests that offering FPDR may also improve psychological outcomes in family members. This calls into question the exclusion of family members as the default option during resuscitations. Further high-quality RCTs in other hospital settings, with longer follow-up of outcomes in both patients receiving resuscitation and family members who have been offered FPDR would be useful to determine the long-term effects of this practice on both patient survival and psychological outcomes in family members.

## Additional file

Additional file 1:
**Sample of electronic search strategies.** (DOCX 497 kb)

## Abbreviations

CPR: cardiopulmonary resuscitation
ER: emergency room
FPDR: family presence during resuscitation
GRADE: Grading of Recommendations Assessment, Development and Evaluation
ICU: intensive care unit
ITT: intention-to-treat
PTSD: post-traumatic stress disorder
RCT: randomized controlled trial

## References

[1] Cooper JA, Cooper JD, Cooper JM (2006). Cardiopulmonary resuscitation: history, current practice, and future direction. Circulation.

[2] Doyle CJ, Post H, Burney RE, Maino J, Keefe M, Rhee KJ (1987). Family participation during resuscitation: an option. Ann Emerg Med.

[3] Downar J, Kritek PA (2013). Family presence during cardiac resuscitation. N. Engl. J. Med.

[4] McAlvin SS, Carew-Lyons A (2014). Family presence during resuscitation and invasive procedures in pediatric critical care: a systematic review. Am J Crit Care.

[5] Porter J, Cooper SJ, Sellick K (2013). Attitudes, implementation and practice of family presence during resuscitation (FPDR): a quantitative literature review. Int Emerg Nurs.

[6] Higgins JP, Altman DG, Gøtzsche PC, Jüni P, Moher D, Oxman AD (2011). The Cochrane Collaboration’s tool for assessing risk of bias in randomised trials. BMJ.

[7] Hozo S, Djulbegovic B, Hozo I (2005). Estimating the mean and variance from the median, range, and the size of a sample. BMC Med Res Methodol.

[8] Balshem H, Helfand M, Schunemann HJ, Oxman AD, Kunz R, Brozek J (2011). GRADE guidelines: 3. Rating the quality of evidence. J Clin Epidemiol.

[9] Jabre P, Belpomme V, Azoulay E, Jacob L, Bertrand L, Lapostolle F (2013). Family presence during cardiopulmonary resuscitation. N. Engl. J. Med.

[10] Robinson SM, Mackenzie-Ross S, Hewson GL (1998). Psychological effect of witnessed resuscitation on bereaved relatives. The Lancet.

[11] Holzhauser K, Finucane J, De Vries SM (2006). Family presence during resuscitation: a randomised controlled trial of the impact of family presence. Australas. Emerg. Nurs. J.

[12] Dudley NC, Hansen KW, Furnival RA, Donaldson AE, Van Wagenen KL, Scaife ER (2009). The effect of family presence on the efficiency of pediatric trauma resuscitations. Ann Emerg Med.

[13] Weiss DS, Marmar CR (1997). “The impact of event scale-revised.” Assessing psychological trauma and PTSD.

[14] Zigmond AS, Snaith RP (1983). The Hospital Anxiety and Depression Scale. Acta Psychiatr Scand.

