# Supplementary material for: The offering of family presence during resuscitation: a systematic review and meta-analysis
Source: J Intensive Care. 2015 Oct 14;3:41. doi: 10.1186/s40560-015-0107-2 (PMC4607174; doi:10.1186/s40560-015-0107-2)
Supplement: Additional file 1: — Sample of electronic search strategies. (DOCX 497 kb) [file 40560_2015_107_MOESM1_ESM.docx]

**Additional File 1: Sample of electronic search strategies**

**Ovid MEDLINE(R)** 1946 to September 2015

1. heart arrest/ or death, sudden, cardiac/ or out-of-hospital cardiac arrest/

2. respiratory insufficiency/ or acidosis, respiratory/ or airway obstruction/

3. emergency treatment/ or advanced trauma life support care/ or resuscitation/ or cardiopulmonary resuscitation/ or advanced cardiac life support/ or heart massage/ or respiration, artificial/ or resuscitation orders/

4. emergency service, hospital/ or trauma centers/

5. Intensive Care/mt, og, px [Methods, Organization & Administration, Psychology]

6. resus*.mp.

7. (cardiopulmonary or CPR).mp. [mp=title, abstract, original title, name of substance word, subject heading word, keyword heading word, protocol supplementary concept, rare disease supplementary concept, unique identifier]

8. or/1-7

9. *Family/px [Psychology]

10. (family adj3 presen*).mp. [mp=title, abstract, original title, name of substance word, subject heading word, keyword heading word, protocol supplementary concept, rare disease supplementary concept, unique identifier]

11. family.mp.

12. or/9-11

13. 8 and 12

1. limit 13 to (humans and clinical trial, all)

**Cochrane Central Register of Controlled Trials** September 2015

1. heart arrest/ or death, sudden, cardiac/ or out-of-hospital cardiac arrest/

2. respiratory insufficiency/ or acidosis, respiratory/ or airway obstruction/

3. emergency treatment/ or advanced trauma life support care/ or resuscitation/ or cardiopulmonary resuscitation/ or advanced cardiac life support/ or heart massage/ or respiration, artificial/ or resuscitation orders/

4. emergency service, hospital/ or trauma centers/

1. Critical care/ or intensive care/

6. intensive care.mp.

7. resus*.mp.

8. (cardiopulmonary or CPR).mp. [mp=title, abstract, original title, name of substance word, subject heading word, keyword heading word, protocol supplementary concept, rare disease supplementary concept, unique identifier]

9. or/1-8

10. Family Therapy/ or Family/ or family.mp. or Family Conflict/

11. (family adj3 presen*).mp. [mp=title, abstract, original title, name of substance word, subject heading word, keyword heading word, protocol supplementary concept, rare disease supplementary concept, unique identifier]

12. or 10-11

1. 9 and 12
2. limit 13 to limit 13 to (clinical trial or clinical trial, phase i or clinical trial, phase ii or clinical trial, phase iii or clinical trial, phase iv or controlled clinical trial or randomized controlled trial)
